# Internalization and Intoxication of Human Macrophages by the Active Subunit of the *Aggregatibacter actinomycetemcomitans* Cytolethal Distending Toxin Is Dependent Upon Cellugyrin (Synaptogyrin-2)

**DOI:** 10.3389/fimmu.2020.01262

**Published:** 2020-06-16

**Authors:** Kathleen Boesze-Battaglia, Anuradha Dhingra, Lisa M. Walker, Ali Zekavat, Bruce J. Shenker

**Affiliations:** ^1^Department of Biochemistry, University of Pennsylvania School of Dental Medicine, Philadelphia, PA, United States; ^2^Department of Pathology, University of Pennsylvania School of Dental Medicine, Philadelphia, PA, United States

**Keywords:** cytolethal distending toxin, *Aggregatibacter actinomycetemcomitans*, macrophages, bacterial toxins, cellugyrin, lipid rafts

## Abstract

The *Aggregatibacter actinomycetemcomitans* cytolethal distending toxin (Cdt) is a heterotrimeric AB_2_ toxin capable of inducing cell cycle arrest and apoptosis in lymphocytes and other cell types. Recently, we have demonstrated that human macrophages are resistant to Cdt-induced apoptosis but are susceptible to toxin-induced pro-inflammatory cytokine response involving activation of the NLRP3 inflammasome. Exposure to Cdt results in binding to the cell surface followed by internalization and translocation of the active subunit, CdtB, to intracellular compartments. Internalization involves hijacking of retrograde pathways; treatment of cells with Retro-2 leads to a decrease in CdtB–Golgi association. These events are dependent upon toxin binding to cholesterol in the context of lipid rich membrane microdomains often referred to as lipid rafts. We now demonstrate that within 1 h of exposure of macrophages to Cdt, CdtB is internalized and found primarily within lipid rafts; concurrently, cellugyrin (synaptogyrin-2) also translocates into lipid rafts. Further analysis by immunoprecipitation indicates that CdtB associates with complexes containing both cellugyrin and Derlin-2. Moreover, a human macrophage cell line deficient in cellugyrin expression (THP-1^Cg−^) challenged with Cdt failed to internalize CdtB and was resistant to the Cdt-induced pro-inflammatory response. We propose that lipid rafts along with cellugyrin play a critical role in the internalization and translocation of CdtB to critical intracellular target sites in human macrophages. These studies provide the first evidence that cellugyrin is expressed in human macrophages and plays a critical role in Cdt toxicity of these cells.

## Introduction

Cytolethal distending toxins (Cdt) are a family of protein exotoxins produced by over 30 γ- and ε-Proteobacteria; these human and/or animal pathogens share the common property of an ability to colonize mucocutaneous tissue (1, 2). Moreover, recent studies suggest that Cdts contribute to the persistence, invasiveness and severity of disease associated with Cdt-producing organisms (3–8). Key to the toxicity of these exotoxins is the requirement to facilitate internalization of the active Cdt subunit to intracellular compartments. In this regard, most Cdts, including the *Aggregatibacter actinomycetemcomitans* Cdt are heterotrimeric holotoxins that function as AB_2_ toxins. In this toxin model, the CdtA and CdtC subunits serve as the binding complex (B) and CdtB as the internalized active subunit (A) [reviewed in (1, 9)]. In order to deliver CdtB to intracellular compartments the holotoxin must first bind to target cell surfaces. Several investigators have demonstrated that the CdtC subunit of several Cdts, including the *A. actinomycetemcomitans* Cdt, bind to membrane cholesterol (10–17). Cholesterol binding in the context of membrane microdomains was demonstrated utilizing both model membranes and live cells including both lymphocytes and macrophages (10–12). Binding was shown to be dependent upon an amino acid sequence, the cholesterol recognition amino acid consensus sequence (CRAC) encoded within the CdtC subunit. A similar cholesterol recognition unit is present within CdtB and is required for internalization of this subunit (12). Currently, the exact role for CdtA in toxin binding is unclear. CdtA shares structural homology with lectin-like proteins; studies have suggested that fucose moieties as well as glycosphingolipids might be involved in the interaction of this subunit with the cell surface (13, 18–20).

Once CdtB is internalized, it must reach intracellular compartment(s) to intoxicate cells. To date, the identification of the specific intracellular compartment(s) is unclear and is likely dependent on CdtB's mode of action. In this regard it has been demonstrated that CdtB exhibits two enzymatic activities: DNase and lipid phosphatase (1, 21–24). In the case of the former activity, it is believed that the active subunit must translocate to the nucleus where it induces double-strand DNA breaks. In contrast, the lipid phosphatase activity, specifically phosphatidylinositol (PI) 3,4,5-triphosphate (PIP3) phosphatase activity requires that CdtB transits to intracellular pools of PIP3 where it is capable of depleting cells of this lipid signaling molecule and inducing PI-3K signaling blockade (24). PIP3 pools exist in proximity to the internal leaflet of the plasma membrane as well as in association with other intracellular membranes including transport vesicles (25, 26). Thus, internalization of CdtB and translocation to requisite intracellular compartments is likely dependent upon its mode of action which in turn is likely determined by the bacterial source of Cdt as well as the specific target cell. Regardless of the intracellular site, it has been demonstrated that CdtB traffics by retrograde transport to the Golgi and the endoplasmic reticulum (ER) (27, 28).

In recent studies, we (29) and Carette et al. (30, 31) have identified a link between Cdt toxicity and a host cell protein cellugyrin (synaptogyrin-2). Furthermore, we have demonstrated that in lymphocytes, cellugyrin plays a key role in CdtB internalization. Specifically, we have demonstrated that soon after exposure to Cdt, lymphocytes exhibit translocation of the host cell protein cellugyrin (synaptogyrin-2) to the same cholesterol rich microdomains in which CdtB is observed to initially accumulate. In addition to co-localization, it was determined through immunoprecipitation studies that CdtB and cellugyrin are part of the same complex. Moreover, reduced expression of cellugyrin protected cells from Cdt-mediated toxicity: cell cycle arrest and apoptosis (29). In this study, we extend our initial observations to determine if cellugyrin is also critical to CdtB translocation and toxicity in human macrophages.

## Materials and Methods

### Reagents and Antibodies

The following antibodies were obtained from Cell Signaling Technology (Danvers, MA): rabbit anti-EEA1 mAB, rabbit anti-RCAS1 mAb, rabbit anti-AIF mAb and rabbit anti-LAMP1 mAb. Mouse anti-CdtB mAb conjugated to AlexaFluor 488 was generated in our laboratory (see below) and mouse anti-rabbit IgG AlexaFluor 594 was purchased from Invitrogen (Carlsbad, CA). Western blot and immunoprecipitation studies utilized the following antibodies: anti-CdtB mAb, anti-CdtC mAb, and anti-cellugyrin antibody which were generated in our laboratory as previously described (10, 29); additional antibodies include: rabbit anti-derlin-2 (Sigma Aldrich, St. Louis, MO), goat anti-mouse Ig-HRP conjugate, goat anti-rabbit Ig-HRP conjugate (Southern Biotech; Birmingham, AL), mouse anti-transferrin receptor (TfR) mAb (BD Biosciences; San Jose, CA), and rabbit anti-caveolin anti-sera (Sigma Aldrich). Additional reagents include: Retro-2 (Sigma Aldrich) and cholera toxin B (CTB) conjugated to AlexaFluor 594 (Invitrogen).

Construction and expression of the plasmid containing the *cdt* genes for the Cdt holotoxin (pUCAacdtABChis) have previously been reported (11). The histidine-tagged holotoxin was isolated by nickel affinity chromatography. Experiments conducted in this report employed Cdt at the following concentrations: 8 ng/m (0.157 mM), 40 ng/ml (0.784 mM), 200 ng/ml (3.9 mM), and 1 μg/ml (19.5 mM).

### Cell Culture and Lentiviral Transfection

The human acute monocytic leukemia cell line, THP-1, obtained from ATCC were maintained in RPMI 1640 containing 10% FBS, 1 mM sodium pyruvate, 20 μM 2-mercaptoethanol and 2% penicillin-streptomycin at 37°C with 5% CO_2_ in a humidified incubator. THP-1 cells stably expressing shRNA against cellugyrin (THP-1^Cg−^) and control cells (THP-1^shctl^) were prepared with commercially available Lentiviral particles (Santa Cruz Biotech; Dallas, TX). The cells were prepared by transducing THP-1 cells with Lentiviral particles, along with non-target controls, as previously described (32, 33). Expression levels were assessed by Western blot. THP-1 cells were differentiated into macrophages by incubating cells in the presence of 50 ng/ml PMA for 48 h at which time the cells were washed and incubated an additional 24 h in medium prior to use.

### Isolation of Triton X-100 Resistant Membrane Rafts

Triton X-100 resistant membrane rafts were prepared from THP-1^WT^ cells that were incubated in the presence of medium or Cdt holotoxin for 2 h. Following the incubation period, cells were harvested, washed and lysed in the presence of Triton X-100 as previously described (10). The lysates were further fractionated on a density gradient using a commercially available kit (Sigma) as previously described (10). Lysates were layered onto the gradients and centrifuged at 200,000× G for 4 h at 4°C. Two prominent bands were recovered, designated DRM1 and DRM2; these were washed and resuspended in 0.2 ml HEPES buffer. Additionally, two soluble fractions, designated S1 and S2 were obtained from the bottom two 0.5 ml fractions.

### Immunoprecipitation and Western Blot Analysis

Cells were treated as described above and solubilized in 20 mM Tris-HCl buffer (pH7.5) containing 150 mM NaCl, 1 mM EDTA, 1% NP-40, 1% sodium deoxycholate and protease inhibitor cocktail (ThermoFisher Scientific; Waltham, MA). Samples (30 μg) were separated on 12% SDS-PAGE and then transferred to PVDF membranes. The membrane was blocked with BLOTTO and then incubated with one of the primary antibodies (as indicated) for 18 h at 4°C as previously described (29). Membranes were washed and incubated with goat anti-mouse immunoglobulin conjugated to horseradish peroxidase (Southern Biotech Technology; Birmingham, AL). The Western blots were developed using chemiluminescence and analyzed by digital densitometry (Li Cor Biosciences; Lincoln, NE) as previously described (34).

Immunoprecipitation studies were performed with antibody to cellugyrin or CdtB which were immobilized by crosslinking to protein A/G using the Pierce Crosslink IP kit (ThermoFisher Scientific) as previously described (29). THP-1 cells were incubated with medium or Cdt (2 μg/ml) for 2 h at 37°C and then lysed and centrifuged at 5,000× g for 5 min. Supernatants were loaded onto columns containing the protein A/G with cross-linked antibody and incubated overnight at 4°C. The columns were washed and the immunoprecipitated protein eluted and analyzed by Western blot as described above. Western blots were probed using anti-cellugyrin antibody, anti-Cdt subunit mAb or anti-derlin-2 followed by reporter antibody conjugated to HRP.

### Confocal Microscopy

Analysis of cellugyrin association with membrane microdomains was performed as follows. Differentiated THP-1 cells grown on glass bottom dishes were incubated with or without Cdt holotoxin (2 μg/ml) for 1 h at 37°C, and washed twice with medium. Membrane lipid raft staining was performed by incubating the cells with 1 μg/ml inactive cholera toxin B (CTB) conjugated to AlexaFluor 594 for 30 min at 4°C. The cells were washed in medium (without serum), incubated with mouse anti-CTB antibody at 4°C for 30 min followed by 37°C for 30 min, washed and fixed in cold methanol (−20°C) for 15 min, washed again and processed for immunostaining with anti-cellugyrin antibody. Briefly, the cells were blocked in blocking solution containing 5% BSA and 0.2% Triton X-100 in PBS (PBST) at 37°C for 1 h, incubated with anti-cellugyrin antibody conjugated to AlexaFluor488 diluted in blocking solution, and Hoechst 33258 at 4°C overnight, washed three times with PBST. Images were captured on a Nikon A1R laser scanning confocal microscope with a 100× (oil) objective at 18°C, and the data were analyzed using Nikon Elements AR 4.30.01 software. To estimate cellugyrin associated with raft, intensity thresholds were applied to the two channels (green and red) for the control and Cdt treated cells in each experiment; binary images were used to determine intersection area between cellugyrin (green) and raft (red) stained structures in each field. Intersection area was then normalized to total cellugyrin positive area for comparisons across experiments. Two-tailed, one sample *T*-test was used to test if mean cellugyrin associated with lipid rafts (normalized to total cellugyrin) in Cdt treated cells relative to control cells was different from 1.

Trafficking studies were performed as follows. Differentiated THP-1^WT^ or THP-1^Cg−^ cells grown on glass bottom dishes were incubated with 2 μg/ml Cdt holotoxin at 4°C for 30 min to facilitate binding, followed by 37°C for 45 min to stimulate uptake. Cells were subsequently washed in PBS and fixed in 4% PFA for 15 min at RT. For retrograde inhibition experiments, differentiated THP-1^WT^ cells grown on glass bottom dishes were treated with Retro-2 (100 μM) or in medium (control) at 37°C for 30 min, followed by addition of 1 μg/ml Cdt holotoxin and incubation at 4°C for 30 min and then at 37°C for 45 min in the continued presence of Retro-2 (100 μM). Cells were washed in PBS and fixed in 4% PFA for 15 min at RT. The cells were immunostained for the CdtB subunit and intracellular markers as described below for immunostaining.

Immunostaining was performed on PFA fixed cells that were permeabilized and blocked in 5% BSA and 0.2% Triton X-100 in PBS (PBST) at 37°C for 1 h. Cells were then incubated with the primary antibody diluted in blocking solution at 4°C overnight, washed three times with PBST, incubated with appropriate secondary antibodies conjugated to AlexaFluor 594 and Hoechst 33258 at 37°C for 1 h and washed three times. Immunostaining was performed using anti-CdtB mAb conjugated to AlexaFluor 488 antibody along with different intracellular marker antibodies: EEA1, RCAS1; AIF; LAMP1. Images were captured on a Nikon A1R laser scanning confocal microscope with a 100× (oil) objective at 18°C, and the data were analyzed using Nikon Elements AR 4.30.01 software. To estimate the levels of CdtB subunit associated with golgi, golgi stained structures (red) were identified by drawing regions of interest (ROI) and sum intensity of CdtB stained structures (green) within each ROI was estimated and plotted as a percentage of total CdtB in the field for both control and Retro-2 treated samples.

### Flow Cytometric Analysis of Cdt Binding and CdtB Internalization

Cdt binding and CdtB internalization was detected by incubating THP-1^WT^ and THP-1^Cg−^ macrophages for 30 min (surface staining; 5°C) or 1 h (intracellular staining; 37°C) in the presence of medium or 2 μg/ml of Cdt. Surface Cdt was assessed by staining cells with anti-CdtC (or anti-CdtB) conjugated to AlexaFluor 488 followed by flow cytometric analysis as previously described (11). Briefly, cells were washed, exposed to normal mouse IgG (Zymed Labs; San Franscisco, CA) and then stained (30 min) for the Cdt subunit with anti-CdtC (or anti-CdtB) subunit mAb conjugated to AlexaFuor 488 (Molecular Probes; Eugene, OR). Following fixation with 2% paraformaldehyde the cells were analyzed by flow cytometry. Intracellular CdtB was detected after exposure of cells to toxin (or medium) and fixation with 2% formaldehyde for 30 min followed by permeabilization with 0.1% Triton X-100 in 0.1% sodium citrate. Cells were and stained with anti-CdtB mAb conjugated to Alexa Fluor 488. Note that this protocol does not detect surface associated CdtB.

### Measurement of Cytokine Release by ELISA

Cytokine production was measured in THP-1 derived macrophages incubated for 5 h in the presence or absence of varying amounts of Cdt (0–200 ng/ml). Culture supernatants were collected and analyzed by ELISA for IL-1β (Quantikine Elisa Kit; R and D Systems) or TNFα (Peprotech) using commercially available kits according to the manufacturer's instructions. In each instance, the amount of cytokine present in the supernatant was determined using a standard curve.

## Results

**Figure 1:**
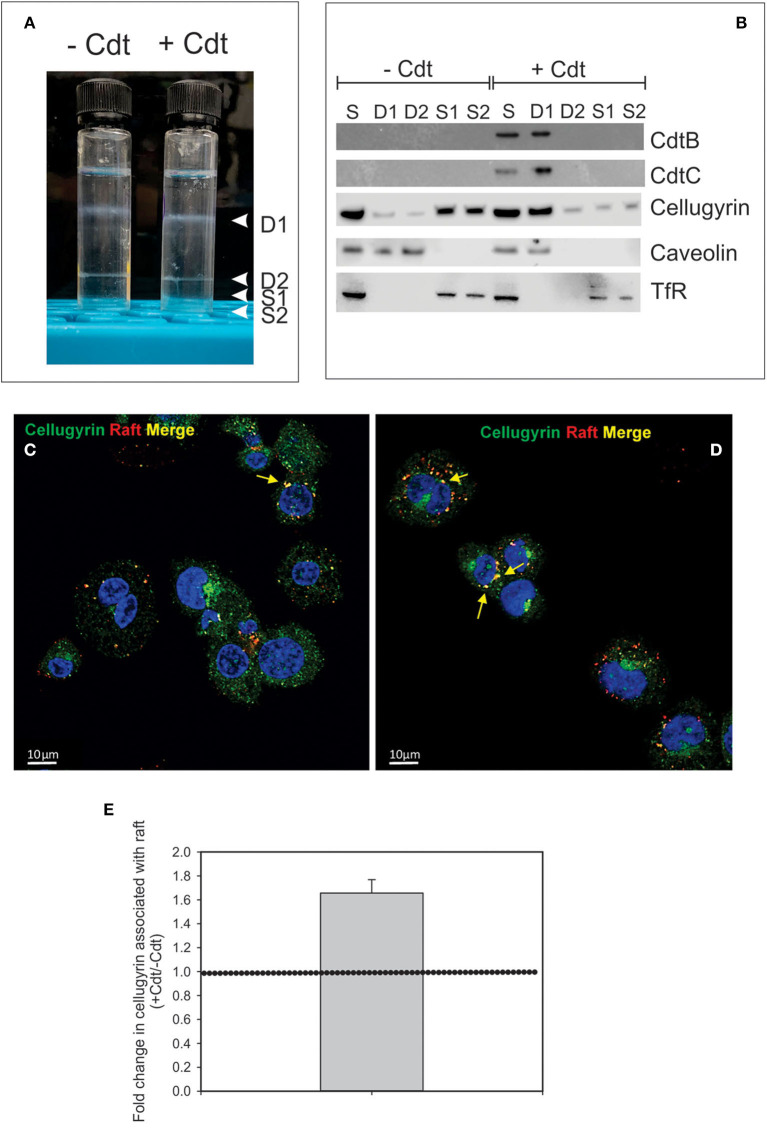
Cdt-induces translocation of cellugyrin to cholesterol rich microdomains. THP-1^WT^-derived macrophage were treated with medium or Cdt (1 μg/ml) for 1 h. Cells were harvested, washed, and cholesterol rich microdomains isolated as detergent resistant membranes (DRM) as described in Materials and Methods. **(A)** Shows the two DRM zones, DRM1 (D1) and DRM2 (D2), on the sucrose gradient; additionally two soluble fractions were collected, designated S1 and S2, which contain the bottom 0.5 ml (each) of the gradient. **(B)** Each fraction was analyzed by Western blot for the presence of CdtB, CdtC, cellugyrin, caveolin (lipid raft marker) and the transferrin receptor (TfR) (non-raft membrane marker). S refers to unfractionated start material. **(C,D)** Representative confocal images of differentiated untreated THP-1^WT^ cells (Ctl; **C**) or treated with 1 μg/ml Cdt (+Cdt; **D**) and stained for rafts (red) and cellugyrin (green). **(E)** Shows the fold change in cellugyrin associated with lipid rafts in Cdt treated cells relative to untreated cells (normalized to total cellugyrin). Dashed horizontal line represents unity. Results are from three experiments (4–7 fields per experiment); *p* <0.05 (two-tailed one sample *t*-test). The degree of overlap between cellugyrin and lipid raft staining was estimated by Pearson's correlation coefficient of 0.72 ± 0.04 and 0.73 ± 0.03 (mean ± SEM), *n* = 10 ROI for control and Cdt-treated cells, respectively.

It is well-established that in order to intoxicate cells, the active Cdt subunit, CdtB, must be internalized and then transported to intracellular compartments. Although there is some disagreement as to which intracellular site(s) CdtB must reach to be effective, it is generally accepted that the subunit first traffics in a retrograde manner through the Golgi into the endoplasmic reticulum (ER). In previous studies we identified three key events that must occur prior to intoxication. First, Cdt association with both lymphocytes and macrophages involves binding to cholesterol in the context of cholesterol rich membrane microdomains (10–12). Second, internalized CdtB in lymphocytes was observed to initially accumulate in these cholesterol rich microdomains (lipid rafts) (10). Third, transport from the membrane to intracellular compartments in lymphocytes was observed to be dependent upon association with a complex containing cellugyrin (synaptogyrin 2), a non-neuronal paralog of the synaptic vesicle protein, synapogyrin 1 (29). Therefore, to advance our understanding of the early events leading to Cdt-induction of pro-inflammatory responses in human macrophages, we first determined if CdtB is also found initially associated with lipid rafts. THP-1^WT^-derived macrophages were treated with Cdt (1 μg/ml) for 60 min; lipid rafts were isolated as detergent resistant membrane (DRM) fractions. As shown in Figure 1A, two prominent DRM bands were identified, DRM1 (D1) and DRM2 (D2); these bands were eluted from the sucrose gradient in fractions corresponding to refractive indexes of 1.359 and 1.374, respectively. Additionally we collected the bottom two 0.5 ml fractions and designated them as detergent soluble fractions, S1 and S2 with refractive indexes of 1.392 and 1.402, respectively. We used these same parameters in our analysis of detergent resistant CdtB containing fractions isolated from lymphocytes (10).

Aliquots of each of these four fractions obtained from both control cells and Cdt-treated cells and were analyzed by SDS-PAGE and Western blot (Figure 1B). Two Cdt subunits, CdtC, the cholesterol binding subunit, and CdtB, the active subunit were found primarily in DRM1. Cellugyrin distribution was also assessed; in control cells (no toxin), cellugyrin was found primarily in the non-lipid raft fractions S1 and S2. However, treatment with Cdt resulted in the translocation of cellugyrin to DRM1. For control purposes, the distribution of caveolin and TfR was also assessed; caveolin, a lipid raft protein, was found in DRM1 and DRM2 in untreated cells, but only in DRM1 in Cdt treated cells. In contrast, TfR, a non-lipid raft protein, was observed in the S1 and S2 fractions in untreated and Cdt treated cells. The translocation of cellugyrin into membrane microdomains was confirmed using fluorescence microscopy. As shown in Figures 1C,D, lipid raft associated cellugyrin fluorescence increased in Cdt treated cells; this translates to 1.6-fold increase in cellugyrin localization to rafts (Figure 1E).

**Figure 2:**
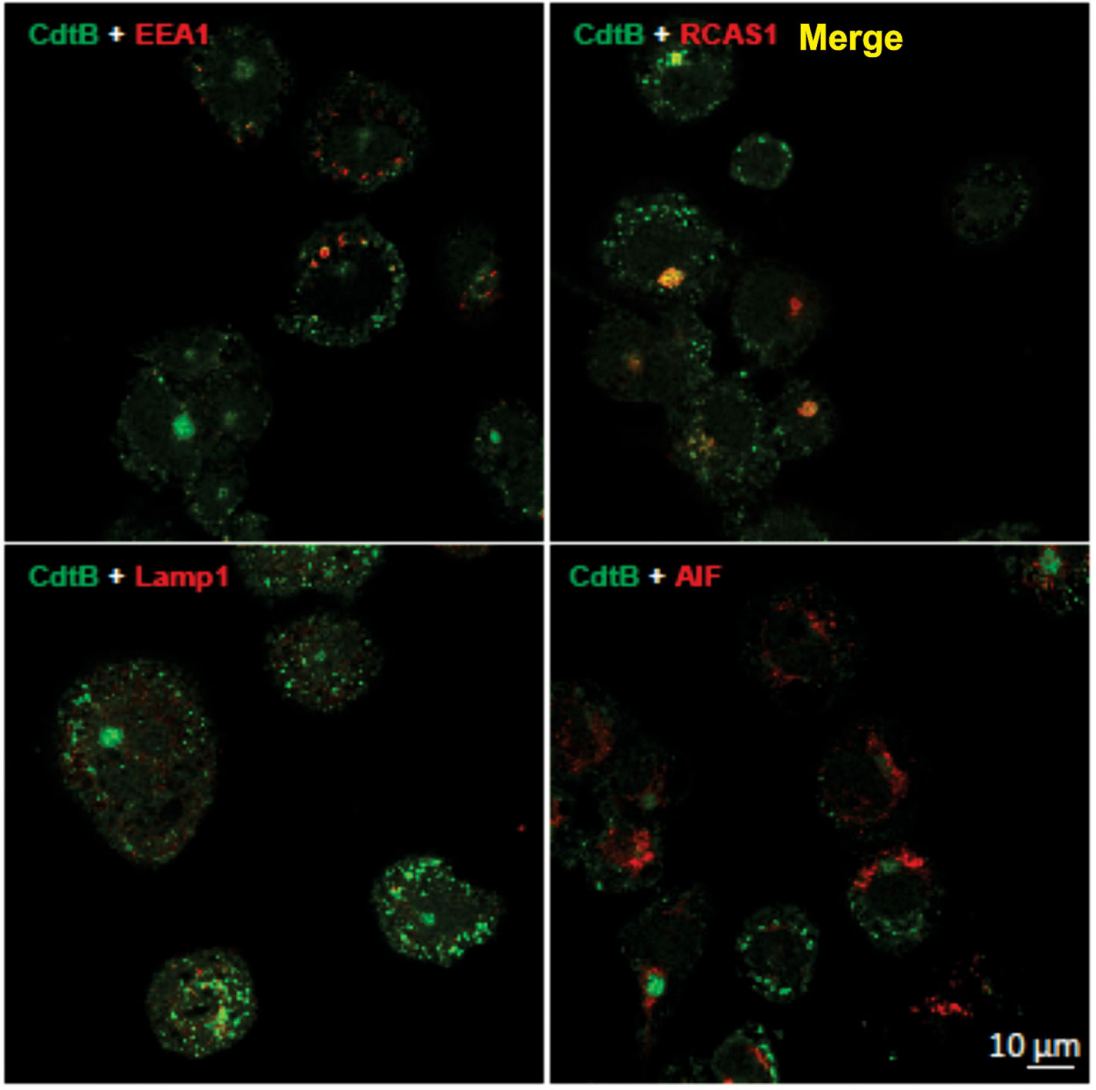
CdtB localizes to early endosomes and Golgi. Confocal micrographs of differentiated THP-1^WT^ cells treated with Cdt holotoxin and immunostained for the indicated organelle markers (red), CdtB (green), and merge (yellow). EEA1, early endosome; RCAS1, Golgi marker; Lamp1, lysosome marker; AIF, mitochondrial marker.

It has been established in several cell types that once internalized, CdtB traffics in a retrograde manner to the Golgi apparatus and on to the ER (9, 27, 28). We first demonstrate that CdtB transport to Golgi also occurs in THP-1^WT^ macrophages (Figure 2). Macrophages treated with Cdt exhibit CdtB fluorescence that is co-localized with early endosomes (EEA1; also see Figure S1) and the Golgi (RCAS1). We did not observe any CdtB fluorescence co-localization with Lamp1, a late endosome marker or mitochondria (AIF); the latter was employed as a control (Figure 2).

**Figure 3:**
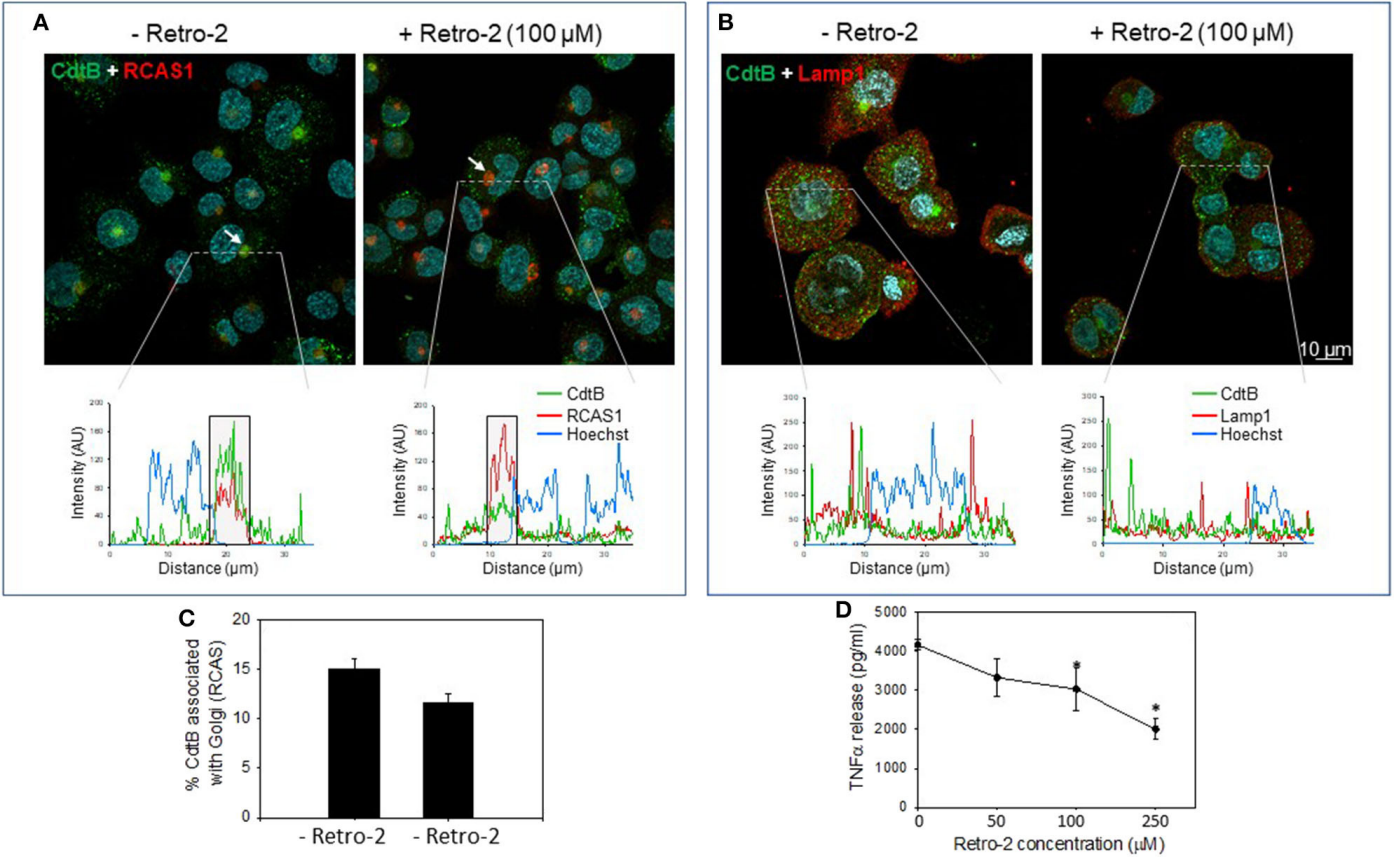
Retro-2 blocks retrograde transport of CdtB to Golgi. Confocal images showing maximum intensity projections (from 6 μn Z stack) of differentiated THP-1^WT^ cells pre-treated for 1 h with medium alone (left) or with Retro-2 (100 μM, right); both samples then received Cdt (1 μg/ml). After 1 h, cells were stained for CdtB and RCAS1 or Lamp1. Nuclei stained with Hoechst are pseudo-colored in cyan. **(A)** Maximum intensity projections for CdtB (green) and RCAS1 (red). Bottom graphs represent intensity profiles for CdtB (green), RCAS1 (red), and Hoechst nuclear stain (blue) across dotted lines depicted. Boxed regions correspond to RCAS1 and CdtB positive structures (marked by white arrows in the top panel). **(B)** Maximum intensity projections for CdtB (green) and Lamp1 (red). Bottom graphs represent intensity profiles for CdtB (green), Lamp1 (red), and nuclear stain (blue) across dotted lines depicted. AU, arbitrary units. All images are representative of four independent experiments (3–4 fields per experiment). **(C)** Shows the results of the analysis for the percent of CdtB associated with Golgi with and without Retro-2. Data are plotted as mean ± SEM. **(D)** THP-1^WT^ macrophages were treated with Retro-2 and Cdt as described above; 5 h later supernatants were analyzed by ELISA for TNFα. Results are the average (pg/ml) TNFα ±SEM of three experiments; *indicates statistical significance (*p* <0.05).

In the next series of experiments, we assessed the requirement for CdtB localization using the inhibitor, Retro-2, a non-toxic inhibitor of endosome-to-Golgi retrograde transport. This inhibitor has been employed in other studies to protect cells from toxins, such as ricin, cholera, and Shiga (35, 36); it also inhibits infection by viruses, such as polyoma, papilloma, AAV, and filoviruses by inhibiting retrograde transport (37–40). Retro-2 has been reported to not alter compartment morphology, endogenous retrograde cargos, or other trafficking steps, such as endocytosis, recycling and degradation (40). CdtB localized to Golgi in the absence of the retrograde transport inhibitor Retro 2 (Figures 3A,B and Figure S2). The linear intensity profile depicts considerable correlation between RCAS1 and CdtB in the absence of Retro-2 (Figure 3A, boxed regions lower panel). Retro-2 treatment resulted in a significant reduction in CdtB associated with Golgi from 15.1 ± 0.01 to 11.6 ± 0.01% (Figures 3A,C). There was no discernible change in Lamp1 and CdtB localization after Retro-2 treatment (Figure 3B). The linear intensity profiles (Figure 3B) suggest that the inhibition of retrograde transport of Cdt does not result in diversion of CdtB to the late endosomal/lysosomal route, as there was little correlation between CdtB and LAMP1 signal. Moreover, macrophages pre-treated with Retro-2 (0–250 μM) exhibited a significant reduction in TNFα release (Figure 3D); untreated control cells did not produce detectable TNFα; in the presence of Cdt (200 ng/ml) alone, cells released 4,165 ± 135 pg/ml TNFα. In contrast cells pre-treated with Retro-2 and Cdt exhibited 3,328 ± 488 pg/ml (50 μM Retro-2), 3,028 ± 544 pg/ml (100 μM Retro-2) and 2,000 ± 266 pg/ml (250 μM Retro-2) TNFα.

**Figure 4:**
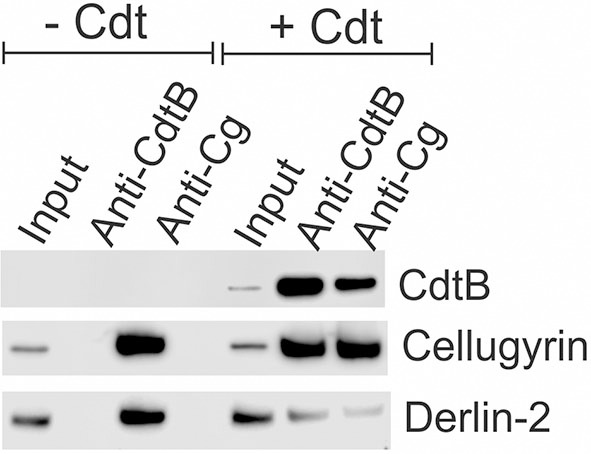
Immunoprecipitation of cellugyrin, Derlin-2, and CdtB. THP-1^WT^ cells were treated with medium or Cdt (2 μg/ml) for 2 h and then washed and homogenized as described in Materials and Methods. Supernatants were immunoprecipitated with either immobilized anti-CdtB or anti-cellugyrin. The bound protein was eluted and analyzed by Western blot for the presence of CdtB, celluygrin, and Derlin-2. Results are representative of three experiments.

As noted above, we have previously demonstrated that CdtB and cellugyrin not only co-localize within lipid rafts, but we have also observed, using both surface plasmon resonance (SPR) and immunoprecipitation, that the two proteins are found associated with one another (29). Therefore, we next employed immunoprecipitation to determine if toxin treatment of macrophages also leads to an association between cellugyrin and CdtB. THP-1^WT^ macrophages were treated with medium alone (control) or with Cdt (2 μg/ml) for 2 h. Cell extracts were prepared and Western blot analyses performed on immunoprecipitates obtained using immobilized anti-CdtB or anti-cellugyrin antibodies. As shown in Figure 4, immunoprecipitation with anti-CdtB pulled down both CdtB and cellugyrin. Immunoprecipitation with anti-cellugyrin antibody pulled down cellugyrin in control cells as well as both cellugyrin and CdtB in the toxin treated cells. In addition to cellugyrin, Eshraghi et al. (27) have recently demonstrated that components of the ER associated degradation (ERAD) machinery are critical to the intracellular transport of CdtB. Therefore, we also assessed the immunoprecipitate for Derlin 2, an ERAD component. As shown in Figure 4, anti-CdtB pulled down Derlin-2 from extracts derived from toxin treated cells. The anti-cellugyrin antibody pulled down Derlin-2 from untreated cells; Derlin-2 was also found in immunoprecipitates of extracts from toxin treated cells, but to a lesser degree than observed with control cells.

**Figure 5:**
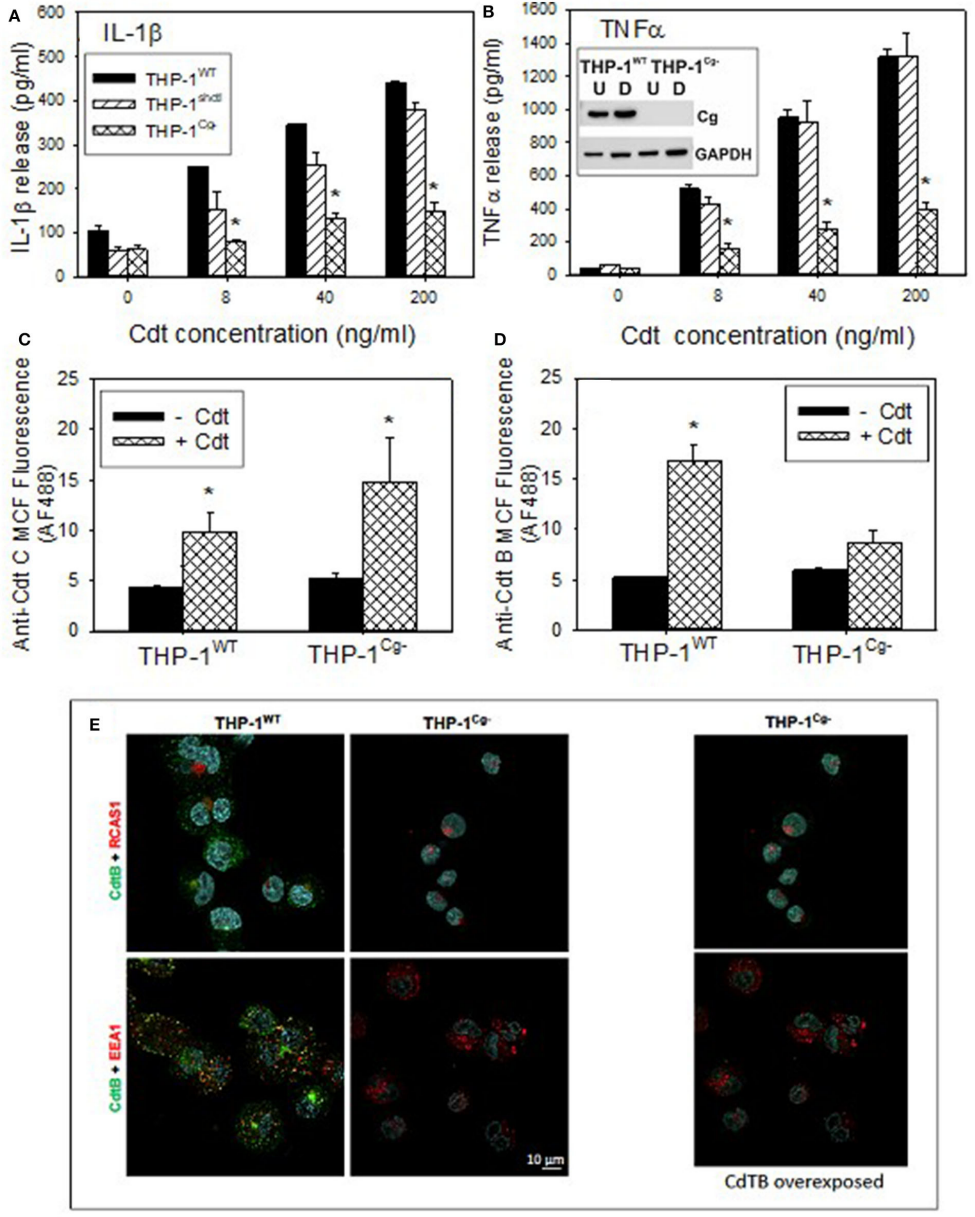
Comparison of THP-1^WT^- and THP-1^Cg−^ -derived macrophages on susceptibility to Cdt-induced pro-inflammatory response, Cdt holotoxin binding and CdtB internalization. **(A,B)** Macrophages derived from THP-1^WT^ (solid bars) THP-1^schctl^ (hatched bars) and THP-1^Cg−^ (cross-hatched bars) cells were treated with varying amounts of Cdt for 5 h; supernatants were harvested and analyzed by ELISA for IL-1β **(A)** and TNFα **(B)**. Data are plotted as the mean ± SEM (pg/ml) and represent the results from three experiments. Inset in **(B)** shows Western blot analysis of cellugyrin in undifferentiated (U) and differentiated (D) cells derived from THP-1^WT^- and THP-1^Cg−^ cells. **(C)** Shows the results of flow cytometric analysis of Cdt binding to THP-1^WT^- and THP-1^Cg−^ -derived macrophages. Cells were incubated for 60 min at 5°C with Cdt (1 μg/ml), washed and stained for the presence of cell surface associated Cdt using anti-CdtC mAb conjugated to AlexaFlour488. The mean channel fluorescence [mcf; (mean±SEM)] for three experiments is shown. **(D)** Shows the flow cytometric analysis of CdtB internalization. THP-1^WT^- and THP-1^Cg−^ -derived macrophages were incubated with Cdt as above except at 37°C. Cells were washed, fixed, permeabilized and stained with anti-CdtB mAb conjugated to AlexaFluor488. The mcf (mean ± SEM) for three experiments is shown. **(E)** Shows the confocal microscopic analysis of CdtB localization to early endosomes or Golgi in THP-1^WT^- and THP-1^Cg−^ cells. Confocal micrographs of Cdt-treated cells immunostained for CdtB (green) and RCAS1 (red, top) or EEA1 (red, bottom). CdtB signal (green) was enhanced in the panel on the right. Nuclei stained with Hoechst are pseudo-colored in cyan.

To advance our understanding of the biological significance of Cdt and cellugyrin association, perhaps within in the context of a macromolecular complex, we next determined if cellugyrin was critical for Cdt association with cells, CdtB internalization and/or toxicity. A cell line deficient in cellugyrin expression (THP-1^Cg−^) was prepared using stable transfection with Lentiviral particles containing cellugyrin specific shRNA (Figure 5B inset). Macrophages derived from the THP-1^Cg−^ cells were first assessed for their susceptibility to induction of a pro-inflammatory cytokine response by Cdt. THP-1^WT^ and THP-1^Cg−^ cells were treated with 0–200 ng/ml Cdt; supernatants were analyzed by ELISA for the presence of IL-1β and TNFα (Figures 5A,B). Cdt-treated THP-1^WT^ cells exhibited dose-dependent increases in IL-1β: 105 pg/ml (0 Cdt), 250 pg/ml (8 ng/ml Cdt), 345 pg/ml (40 ng/ml Cdt), and 438 pg/ml (200 ng/ml). Control transfected cells (THP-1^shctl^) exhibited similar levels of IL-1β release. In contrast THP-1^Cg−^ macrophages produced significantly less IL-1β: 61 pg/ml (0 Cdt), 80 pg/ml (8 ng/ml Cdt), 130 pg/ml (40 ng/ml Cdt), and 149 pg/ml (200 ng/ml Cdt). Similar results were observed for the release of TNFα; THP-1^WT^ cells exhibited a dose dependent increase in this cytokine: 36 pg/ml (0 Cdt), 514 pg/ml (8 ng/ml Cdt), 950 pg/ml (40 ng/ml), and 1,313 pg/ml (200 ng/ml). THP-1^shctl^ cells exhibited a comparable TNFα response. In contrast THP-1^Cg−^ exhibited a significant reduction in TNFα release (relative to THP-1^WT^ and THP-1^shctl^ derived macrophages): 34 pg/ml (0 Cdt), 159 pg/ml (8 ng/ml Cdt), 267 pg/ml (40 ng/ml), and 145 pg/ml (200 ng/ml).

In the last series of experiments we determined if THP-1^Cg−^-derived macrophages were capable of binding Cdt, able to internalize CdtB and/or whether the active subunit trafficked to the endosomes and Golgi. Holotoxin binding to the cell surface was carried out at 5°C and monitored by flow cytometry using anti-CdtC mAb conjugated to AlexaFluor 488 (Figure 5C). THP^WT^ cells were capable of binding toxin as they exhibited mean channel fluorescence (mcf) of 9.7 ± 2.1; control cells exhibited a mcf of 4.3 ± 0.3. Likewise, THP-1^Cg−^ cells retained the capacity to bind Cdt; they exhibited a statistically significant increase in binding over control THP-1^Cg−^ cells with mcfs of 14.7 ± 4.4 (+Cdt) vs. 5.1 ± 0.72 (–Cdt). In addition to utilizing CdtC association with the cell surface as a surrogate of holotoxin binding, we carried out similar analysis for surface association of CdtB (Figure S3). Similar to the results with CdtC, we observed CdtB fluorescence in both THP-1^WT^ and THP-1^Cg−^ cells. It is interesting to note that both assessments indicate greater amounts of toxin (i.e., subunit fluorescence) in macrophages derived from THP-1^Cg−^ cells.

Cells were also assessed for their ability to internalize CdtB (Figure 5D). Following exposure to Cdt at 37°C, cells were evaluated by measuring immunofluorescence with anti-CdtB mAb conjugated to AF488 following fixation and permeabilization. THP-1^WT^ cells exhibited significant CdtB internalization; mcf of Cdt treated cells was 16.7 ± 17 vs. 5.2 ± 0.2 in control cells. In contrast, THP-1^Cg−^ cells did not exhibit significant internalization of CdtB as the mcf was 8.7 ± 1.2 in the presence of Cdt and 5.9 ± 0.3 in untreated cells. It should be noted that in previous studies we have demonstrated that the immunofluorescence due to CdtB internalization observed in THP-1^WT^ cells was dependent on both permeabilization and temperature; cells not permeabilized or those incubated at 5°C failed to exhibit fluorescence when stained with anti-CdtB mAb. The failure of THP-1^Cg−^ cells to internalize CdtB was also confirmed by fluorescence microscopy (Figure 5E).

## Discussion

**Figure 6:**
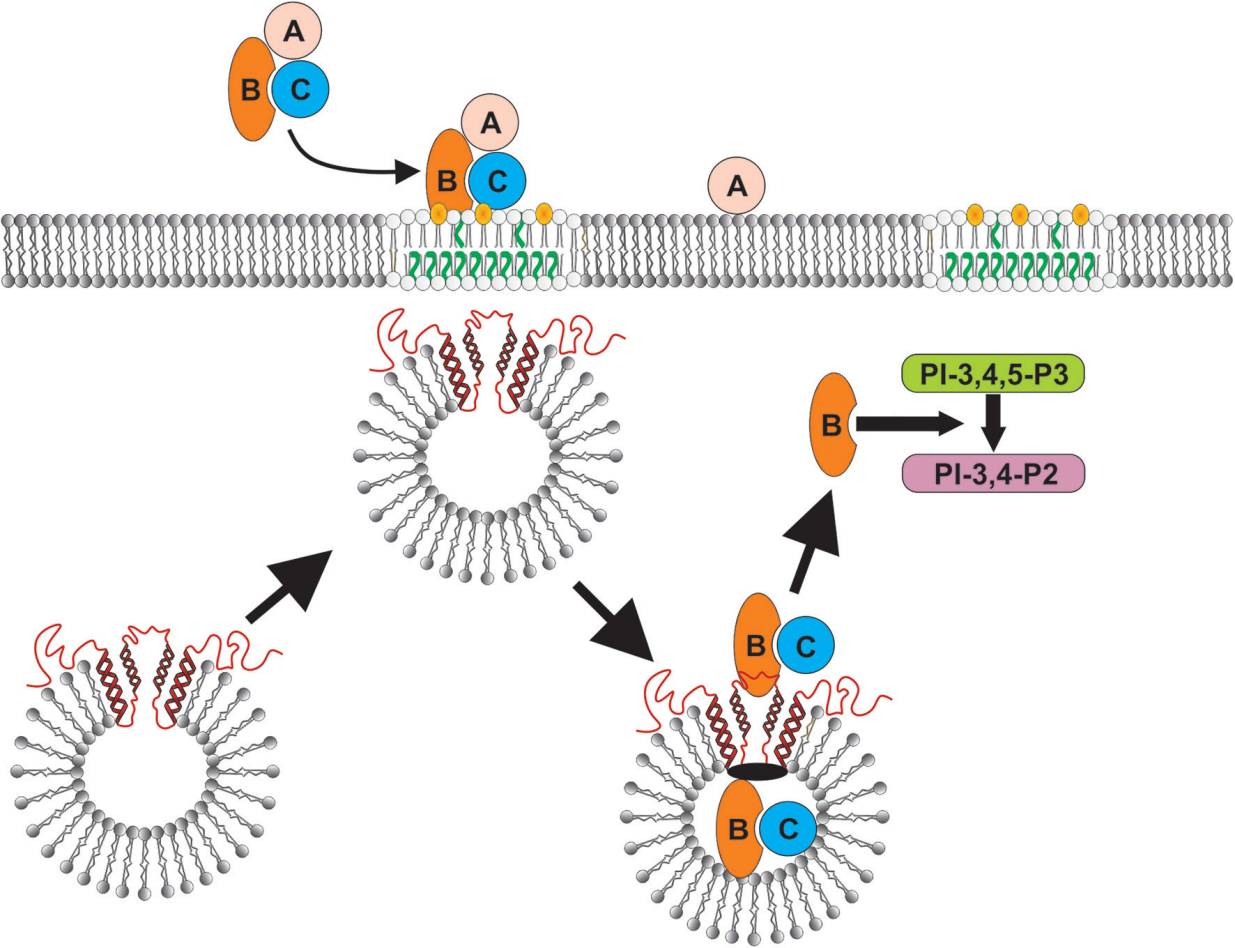
Schematic model showing proposed CdtB-cellugyrin interaction. Cdt holotoxin binds to cells via cholesterol in the context of membrane lipid rafts. CdtB internalization is further dependent upon its ability to interact with cholesterol. As a result of exposure to Cdt, cellugyrin (shown in red) containing SLMVs translocate from cytosol to membrane lipid rafts. We propose that this translocation leads to association of CdtB with the cellugyrin-containing SLMVs. This interaction may involve direct binding to cellugyrin either on extra- or intra-vesicular loops or indirect association via an unidentified binding partner (shown in black). We further propose that CdtB is transported via SLMVs to intracellular target sites; for example sites containing PIP3 pools where the enzymatically active CdtB subunit is released from SLMVs and is then able to degrade the signaling lipid resulting in PI-3K blockade and toxicity.

In this study we demonstrate that the binding of Cdt to human macrophages leads to the association of the active subunit, CdtB, with cholesterol rich membrane microdomains. Exposure to Cdt also leads to the translocation of the host cell protein, cellugyrin, to lipid rafts (summarized in Figure 6). Once internalized, CdtB was found to be part of an intracellular complex that includes cellugyrin and Derlin-2. The expression of cellugyrin, its translocation to lipid rafts and association within a complex containing CdtB and Derlin-2 in human macrophages are novel observations. It should be noted that this relationship was not entirely surprising based upon both our previous studies with Cdt and cellugyrin in lymphocytes (29) and those of Eshraghi et al. (27) who identified a critical role for Derlin-2 as a requisite component of retrograde transport of CdtB. Moreover, macrophages derived from cellugyrin deficient cells (THP-1^Cg−^) retain the capacity to bind Cdt holotoxin, but do not internalize CdtB and are not susceptible to its toxicity which in macrophages is characterized by a pro-inflammatory cytokine response. These findings are consistent with our previous observation that celluygrin is also essential for CdtB internalization and toxicity in human lymphocytes (29).

The ability of Cdt to bind to regions in target cell membranes rich in cholesterol, such as lipid rafts is not surprising. We have previously demonstrated that both CdtC, a component of the toxin binding unit, and CdtB contain CRAC sites which are critical for cholesterol binding and internalization (11); mutation of these sites blocks these events. The significance of Cdt association with cholesterol rich membrane microdomains likely relates to their reported role in serving as regions to concentrate toxins and, more importantly, provide access to endocytic processes (41–43). Indeed, CdtB internalization has been linked to endocytic mechanisms dependent upon dynamin and involving clathrin coated pits (44, 45).

It is now well-established that once internalized, CdtB traffics by retrograde transport from early endosomes to the Golgi and then on to the ER (27, 28, 44–49). Both the transport mechanism and specific location(s) beyond that of the ER is not clear and is likely linked to CdtB's mode of action which is discussed below. Recently, Eshraghi et al. (27) have reported that intracellular trafficking of CdtB beyond the Golgi involves the ERAD pathway. In contrast, other studies suggest that this pathway is not involved in CdtB transport and toxicity. Regardless, we confirm that CdtB must translocate to the Golgi as Retro-2, an inhibitor of endosome-Golgi transport, disrupts CdtB translocation in macrophages; importantly, this blockade also leads to inhibition of cell susceptibility to toxicity.

Our study also demonstrates that cellugyrin must be present for CdtB to be internalized in macrophages. Cellugyrin is a member of a family of proteins known as synaptogyrins which contain four transmembrane regions with a tyrosine-phosphorylated tail (50). Three synaptogyrin isoforms exist. Synaptogyrin 1 and 3 are neuronal and are the most abundant protein in synaptic vesicles; these are critical to vesicle biogenesis, exocytosis and endocytotic recycling as well as neurotransmission (51). Synaptogyrin-2 (cellugyrin) is expressed in all tissue, except brain; it is considered to be a component of synaptic-like microvesicles (SLMVs) (50, 52–54). Cellugyrin, like synaptogyrin-1, is critical for the biogenesis of cellugyrin-containing SLMVs. To date there is no information available that identifies a specific physiologic function(s) associated with these SLMVs in macrophages or for that matter other cell types. Kupriyanaova and Kandror (54) did, however, demonstrate that adipose cells contain cytoplasmic SLMVs that were positive for both cellugyrin and Glut4. The authors were unable to demonstrate that the vesicles localized to the plasma membrane following insulin treatment; nevertheless, it was proposed that these SLMVs represent early sorting vesicles and are likely a component of the trans Golgi network (TGN). Chapel et al. (55) have also proposed that cellugyrin may function as a lysosomal transporter protein.

In addition to a potential role in transport vesicles, there is now increasing evidence that cellugyrin, and in turn, cellugyrin-containing SLMVs is a host target that is hijacked by pathogens for the purposes of intracellular trafficking and retrograde transport. Our studies suggest that cellugyrin plays a critical role in the internalization/transport of CdtB from the cholesterol rich membrane microdomains to intracellular compartments. It has also been shown that viruses exploit these same SLMVs. Sun et al. (56) first reported on the role of cellugyrin in the infection of mammalian cells by *Bunyavirus* which is responsible for severe fever with thrombocytopenia syndrome virus (SFTSV). Cellugyrin was shown to interact with non-structural viral proteins and further, these interactions led to the transport of these proteins into inclusion bodies that were “reconstructed from lipid droplets” during viral infection. The importance of this translocation was demonstrated by silencing cellugyrin expression which resulted in reduced inclusion body formation and viral titers. More recently, Walker et al. (57) reported on *Porcine circovirus 2* (PCV2) which is responsible for a group of diseases known as PCV2 Associated Disease. PCV2 disease exhibits variation in incidence and severity of disease; investigators proposed this variability is due to missense mutation in the SYNGR2 gene (cellugyrin) which results in altered viremia. Experimental silencing of SYNGR expression was found to significantly reduce PCV2 titers in infected PK15 cells. It was proposed that cellugyrin mutation(s) affect its incorporation into vesicular membranes and thereby alter vesicle formation; the altered vesicles fail to transport PCV2 to the nucleus where the virus replicates.

In conclusion, although little is known about the physiologic role of cellugyrin and/or cellugyrin containing SLMVs, it is becoming increasingly clear that they are hijacked by viruses and the bacterial toxin Cdt. In the case of the latter, CdtB internalization and transport to intracellular target sites is dependent upon cellugyrin expression, and likely cellugyrin (and possibly Derlin-2 as well) containing SLMVs (Figure 6). It should be noted that there are currently two paradigms that account for the molecular mechanism by which CdtB induces toxicity in target cells [reviewed in (1, 22)]. First, CdtB has been shown to exhibit low level DNase activity. Several investigators have proposed that this enzymatic activity induces DNA strand breaks leading to toxicity in proliferating cells: cell cycle arrest and apoptosis. There is little direct evidence of DNA damage in Cdt treated mammalian cells; however, investigators have observed that some types of Cdt treated cells exhibit activation of the DNA damage response (DDR) (58–61); this responses utilizes phosphorylation of H2AX as a surrogate for DNA damage. We have proposed an alternative paradigm which is based upon our observation that CdtB, derived from *A. actinomycetemcomitans aggregatibacter* Cdt, is a potent PIP3 phosphatase; Cdt treated cells exhibit depletion of PIP3 and downstream PI-3K blockade: decreased phosphorylation of Akt (inactivation) and GSK3β (activation). Furthermore, we have demonstrated that CdtB toxicity, which we have observed in both proliferating cells and non-proliferating cells, is dependent upon its PIP3 phosphatase activity and not DNase activity (24, 62–65). It is likely that the operative molecular mode of action relates to the amount of toxin employed, the bacterial source of Cdt and the host target cell. Regardless of which mode of action may be operative, it is clear that they both require internalization and retrograde transport of CdtB. Clearly, the final intracellular target site will be closely linked to CdtB's molecular mode of action. Toxicity due to PIP3 depletion would require that CdtB accumulates in close proximity to the intracellular leaflet of plasma membranes and organelle membranes as these are areas in which PIP3 pools are generated. In summary, our current study, in conjunction with our previous findings in lymphocytes, clearly demonstrate that cellugyrin, and likely cellugyrin containing SLMVs, play a requisite role in the earliest stages of internalization and possibly retrograde transport of CdtB from lipid rafts to intracellular target sites. These observations not only advance our understanding of upstream events leading to toxicity, but also identify potential sites for developing protocols for therapeutic intervention to prevent Cdt mediated toxicity and inhibit the pathogenesis of disease caused by Cdt-producing pathogens.

## Data Availability Statement

All datasets generated for this study are included in the article/Supplementary Material.

## Author Contributions

KB-B and BS conceptualized the research goals, designed the study, provided the administrative oversight, acquired the major funding, and wrote the manuscript. AD, LW, and AZ performed the research, analyzed the results, and participated in discussions regarding experimental design, data interpretation, and experimental planning. All authors contributed to the article and approved the submitted version.

## Conflict of Interest

The authors declare that the research was conducted in the absence of any commercial or financial relationships that could be construed as a potential conflict of interest.
