# Supplementary figures and images for: Internalization and Intoxication of Human Macrophages by the Active Subunit of the Aggregatibacter actinomycetemcomitans Cytolethal Distending Toxin Is Dependent Upon Cellugyrin (Synaptogyrin-2)
Source: Front Immunol. 2020 Jun 16;11:1262. doi: 10.3389/fimmu.2020.01262 (PMC7325893; doi:10.3389/fimmu.2020.01262)

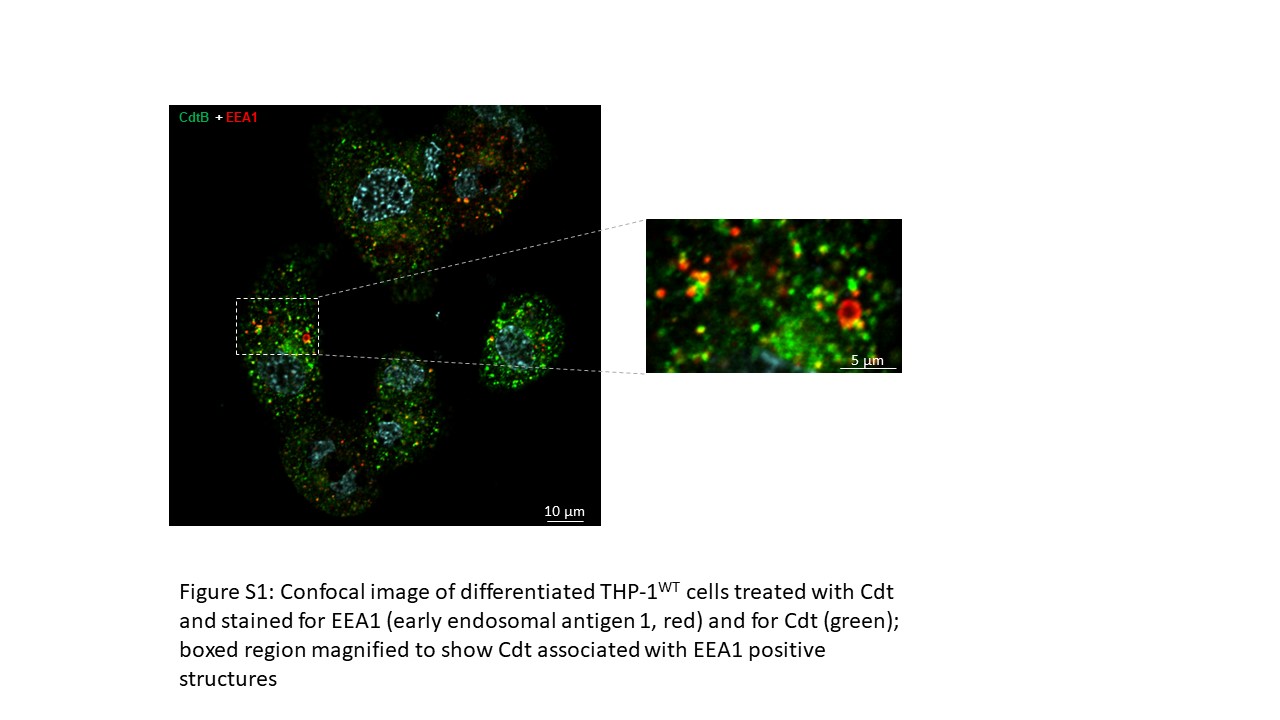

Supplement: Supplementary file 1 [file Image_1.JPEG]

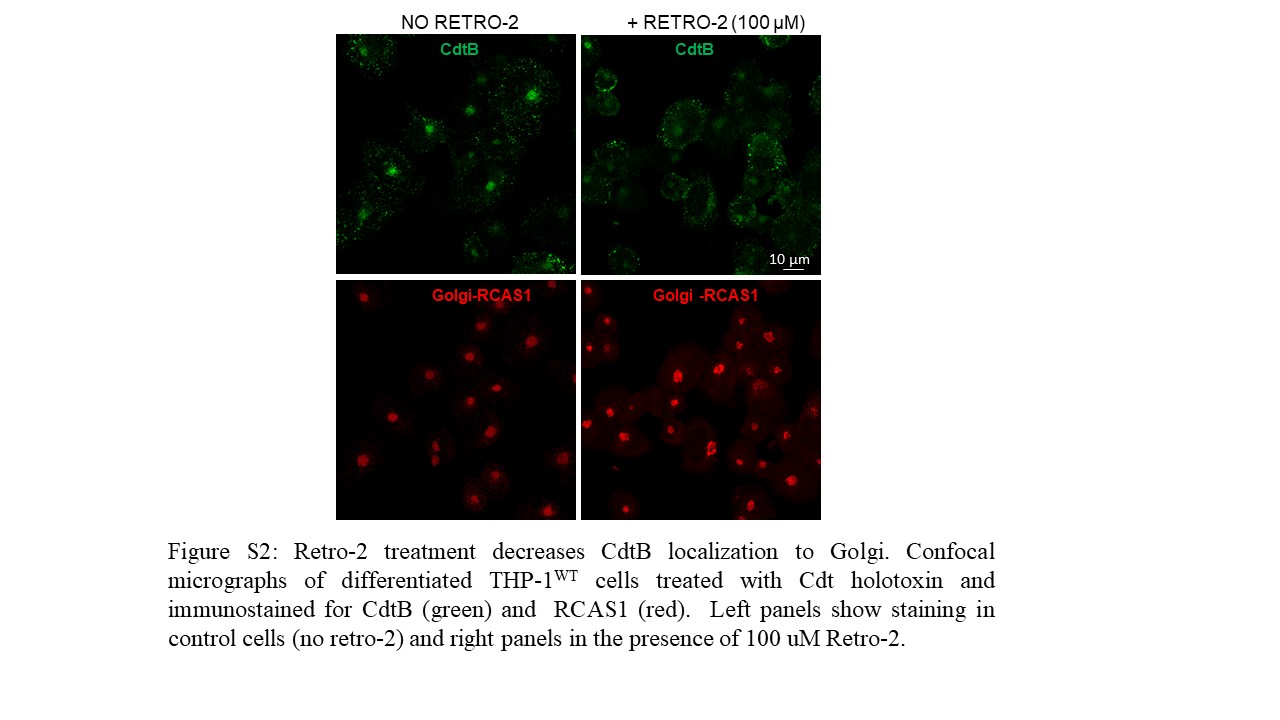

Supplement: Supplementary file 2 [file Image_2.JPEG]

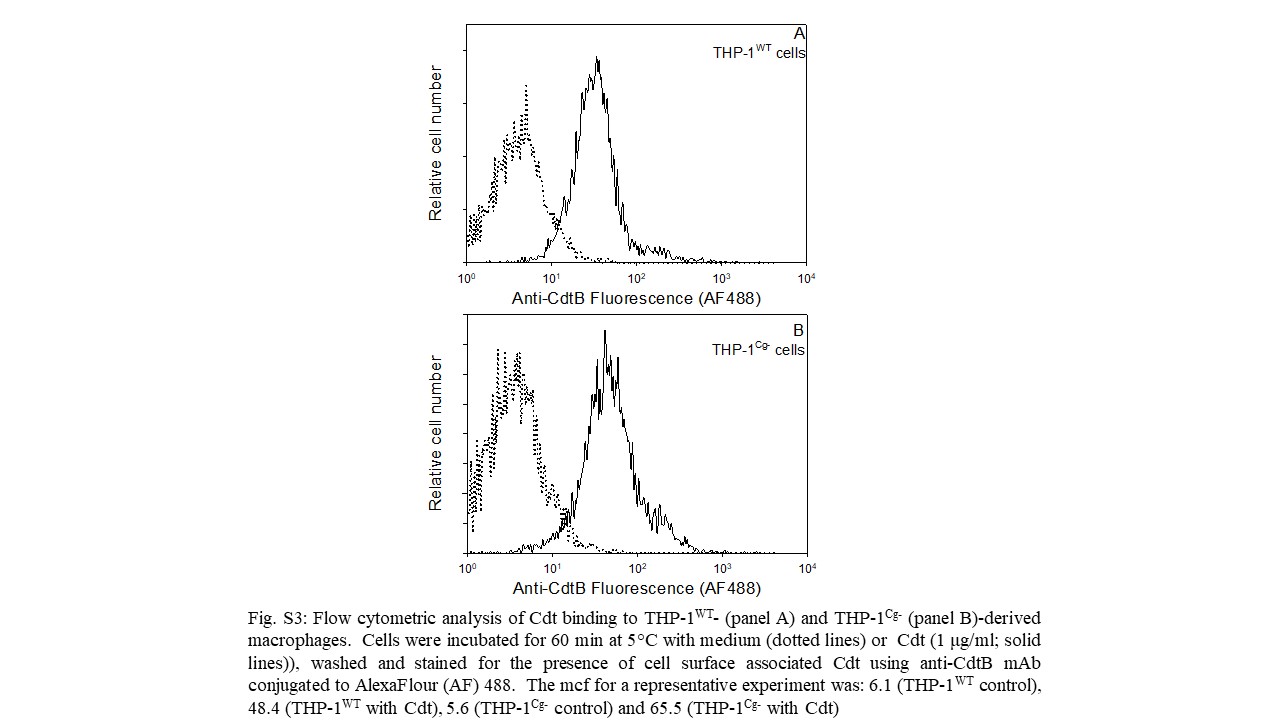

Supplement: Supplementary file 3 [file Image_3.JPEG]
